# Delayed Admission to the Intensive Care Unit and Mortality of Critically Ill Adults: Systematic Review and Meta-analysis

**DOI:** 10.1155/2022/4083494

**Published:** 2022-02-07

**Authors:** Panagiotis Kiekkas, Anastasios Tzenalis, Vasiliki Gklava, Nikolaos Stefanopoulos, Gregorios Voyagis, Diamanto Aretha

**Affiliations:** ^1^Nursing Department, University of Patras, Patras, Greece; ^2^Department of Anesthesiology and Critical Care Medicine, Patras University Hospital, Patras, Greece

## Abstract

Delayed admission of patients to the intensive care unit (ICU) is increasing worldwide and can be followed by adverse outcomes when critical care treatment is not provided timely. This systematic review and meta-analysis appraised and synthesized the published literature about the association between delayed ICU admission and mortality of adult patients. Articles published from inception up to August 2021 in English-language, peer-reviewed journals indexed in CINAHL, PubMed, Scopus, Cochrane Library, and Web of Science were searched by using key terms. Delayed ICU admission constituted the intervention, while mortality for any predefined time period was the outcome. Risk for bias was evaluated with the Newcastle-Ottawa Scale and additional criteria. Study findings were synthesized qualitatively, while the odds ratios (ORs) for mortality with 95% confidence intervals (CIs) were combined quantitatively. Thirty-four observational studies met inclusion criteria. Risk for bias was low in most studies. Unadjusted mortality was reported in 33 studies and was significantly higher in the delayed ICU admission group in 23 studies. Adjusted mortality was reported in 18 studies, and delayed ICU admission was independently associated with significantly higher mortality in 13 studies. Overall, pooled OR for mortality in case of delayed ICU admission was 1.61 (95% CI 1.44-1.81). Interstudy heterogeneity was high (*I*^2^ = 66.96%). According to subgroup analysis, OR for mortality was remarkably higher in postoperative patients (OR, 2.44, 95% CI 1.49-4.01). These findings indicate that delayed ICU admission is significantly associated with mortality of critically ill adults and highlight the importance of providing timely critical care in non-ICU settings.

## 1. Introduction

*Τ*he concept of delayed admission of patients to the intensive care unit (ICU) has attracted international research interest, due to its increasing incidence and its presumed negative impact on patient outcomes [1, 2]. Delayed ICU admission refers to both the waiting time of patients who need critical care in non-ICU settings due to the unavailability of ICU beds and the difficulty of healthcare professionals in identifying timely critical deterioration of patients [3]. The primary reason for increased delayed ICU admission has been the increasing demand for critical care, due to population aging and the increasing number of patients expected to benefit from ICU admission [4, 5]. Other reasons for delayed ICU admission mainly include financial constraints and communication flaws among healthcare professionals [6, 7].

Therapeutic management of critically ill patients is time-sensitive; therefore, delays in the initiation and titration of their treatment could contribute to increased complications and mortality [8–12]. Care of the critically ill in the ICU is expected to offer a survival advantage over non-ICU settings due to the high staff-to-patient ratio, staff expertise, and availability of specialized equipment [10]. In contrast, non-ICU settings commonly used for boarding (that is, waiting until ICU admission) critically ill patients, such as the emergency department (ED) and the postanesthesia care unit (PACU), are not appropriately designed and equipped, while their staff is neither sufficiently trained nor experienced in providing critical care [13, 14]. In this context, delays in the provision of disease-specific protocolized care, including fluid and antibiotic administration, have been reported for critically ill patients boarded in the ED [15, 16].

The intuitive supposition that delayed ICU admission would subject patients to adverse outcomes has been challenged. Critical care is currently not limited to the ICU. Instead, interventions such as noninvasive ventilation and titration of vasopressor drugs are commonly initiated immediately after the identification of critical illness and prior to patient transfer to the ICU [17]. Moreover, sophisticated critical care treatment has become increasingly available in non-ICU settings through the provision of adequate staff training [18]. Therefore, delayed ICU admission might not be necessarily translated into delayed provision of critical care.

The aim of this systematic review and meta-analysis was to identify, appraise, and synthesize qualitatively and quantitatively the existing empirical evidence on the association between delayed ICU admission and mortality of adult patients.

## 2. Methods

### 2.1. Design and Inclusion-Exclusion Criteria

To ensure consistent reporting of findings in this systematic review, guidelines set out in the Preferred Reporting Items for Systematic Reviews and Meta-Analyses (PRISMA) statement were used [19]. The research question was formulated according to the PICO method: “in adult patients (population), what is the mortality (outcome) of patients with delayed ICU admission (intervention) compared with those with timely (nondelayed) ICU admission (comparison)?”

Articles published from inception up to August 31, 2021, in English-language journals were considered for inclusion. Specific inclusion and exclusion criteria were the following:

- Patient population: adult patients admitted to any ICU (medical, surgical, trauma, or mixed). Studies enrolling patients admitted to the pediatric ICU, or critically ill patients not admitted to the ICU, were excluded
- Study design: observational cohort, prospective or retrospective, single- or multicenter
- Intervention: delayed ICU admission, including time periods of any duration until patients were admitted to the ICU after they were considered to require critical care. The delayed ICU admission group consisted of patients who were either not immediately/directly admitted to the ICU or admitted after a particular time period that was considered to constitute delay. These patients were boarded in non-ICU settings, such as the ED, the PACU, and the wards, until ICU admission. Studies in which patients were boarded in subspecialty ICUs (e.g., in the coronary care unit) were excluded
- Outcome: mortality during ICU or hospital stay, or for any predefined time period (e.g., 28-day mortality)
- Reported associations/comparisons: at the univariate level, mortality of the delayed ICU admission group compared with mortality of the nondelayed ICU admission group, which consisted of patients immediately/directly admitted to the ICU, or admitted within a particular time period that was not considered to constitute delay. At the multivariate level, independent associations between delayed ICU admission and mortality were considered. Studies were excluded if delayed and nondelayed ICU admission groups were not defined, or comparisons in mortality between groups were not reported
- Publication types: original full-text articles published in peer-reviewed journals. Dissertations, technical reports, case studies, conference abstracts, and letters were excluded in order to focus on studies that combined detailed information about their methodology and findings with satisfactory methodological quality

### 2.2. Database Search and Study Selection

Search strategy was determined and implemented in consultation with an experienced librarian. Studies indexed in the Cumulative Index for Nursing and Allied Health Literature (CINAHL, via EBSCO), the US National Library of Medicine (MEDLINE, via PubMed), Scopus (via Elsevier), the Cochrane Library (via Wiley), and the Web of Science (via Clarivate Analytics) were searched through an iterative process. The following combinations of free-text search terms were used: “delayed admission”, “admission delay”, “indirect admission”, “delayed transfer”, “boarding”, “emergency department”, “mortality”, “outcome”, “intensive care unit”, “ICU”, and “critically ill”. Medical Subject Headings (MeSH) terms were not used. The detailed literature search strings for each electronic database are presented in Supplementary Materials (available here). Database searches took place in the first week of September 2021.

After searches were completed, retrieved articles were exported into EndNote (X9.3.3 for Windows) for the removal of duplicates. Study selection according to inclusion-exclusion criteria was independently conducted by two authors (AT, NS) in three steps. At the first step, the remaining articles were electronically screened for inclusion according to their titles and abstracts. At the second step, the full text of selected articles was read for determining eligibility for inclusion. At the third step, reference lists of included articles were manually screened to identify additional studies (not found in the online searches). Discrepancies between reviewers were discussed until consensus was reached. The PRISMA flow diagram was used to describe in detail the stepwise study selection process.

### 2.3. Data Extraction, Assessment of Methodological Quality, and Risk for Bias

Two authors (VG, DA) with long expertise in critical care independently extracted data from included studies by using a standardized data collection form, which included

- study characteristics: study design and population, definition of delayed and nondelayed ICU admission groups, incidence of delayed ICU admission, and significant differences in patient characteristics between groups
- study findings: mortality comparisons between delayed and nondelayed ICU admission groups and independent associations between delayed ICU admission and mortality

Risk for bias of the included studies was appraised by the Newcastle-Ottawa Scale (NOS) [20]. For cohort studies, NOS comprises nine items categorized into three groups: selection, comparability, and outcome; therefore, its values range between 0 and 9, with an NOS score ≥ 6 indicating low risk for bias. Each included study was also assessed for seven additional criteria, which would increase risk for bias: single-center design, retrospective design, small population size (<500 patients), exclusion criteria not reported, nondelayed group consisting of patients not immediately admitted to the ICU (but with shorter ICU admission delay than those of the delayed group), significant differences in patient characteristics between groups not reported, and multivariate associations between delayed ICU admission and mortality not reported. Since one point was attributed for each of these criteria, risk for bias ranged between 0 (no risk) and 7 (highest risk). In case data extraction or assessment of the risk for bias was discordant between reviewers, articles were reexamined until discrepancies were resolved by consensus.

### 2.4. Data Synthesis and Analysis

Study characteristics and findings, and assessment of risk for bias, were presented in tables and summarized within the text. Quantitative synthesis of study findings was conducted by using R version 3.6.2 (R Foundation for Statistical Computing). The adjusted odds ratio (OR) for mortality according to delayed ICU admission was used when reported in the study; otherwise, unadjusted OR was used. Hospital mortality was preferred for studies that reported more than one mortality term, followed by 30-day, 28-day, ICU mortality, or any other term used. Likewise, when more than one definition of delayed ICU admission was reported, OR regarding the delay of the longest duration was preferred. Pooled ORs with 95% confidence intervals (CIs) were calculated, and forest plots were constructed to visualize individual and pooled estimates. A common effect size could not be assumed for included studies due to diverse patient populations enrolled, various definitions of delayed and nondelayed ICU admission, and different mortality terms used. Therefore, a random effects approach was preferred, since it is considered to be more conservative and decrease the likelihood of type II errors [21]. Heterogeneity across studies was evaluated by calculating the *I*^2^ statistic. Low, moderate, and high heterogeneities were defined by 25%, 50%, and 75% cut-off *I*^2^ values, respectively.

Subgroup analysis was conducted to assess the validity of findings among patient populations (patients admitted from the ED vs. patients admitted from the wards vs. postoperative patients). Sensitivity analysis was also conducted to investigate potential sources of heterogeneity for the definitions of nondelayed ICU admission (patients immediately/directly admitted to the ICU vs. those with shorter ICU admission delay than that of the delayed ICU admission group) and mortality (adjusted vs. unadjusted, hospital vs. ICU). Publication bias was assessed by constructing a funnel plot, in which the vertical axis represented study size (standard error) and the horizontal axis represented effect size (log risk ratio), and by using Egger's test for evaluating small-study effects. Quality of evidence was evaluated according to the GRADE system criteria by the use of GRADEpro online software [22].

## 3. Results

### 3.1. Study Selection Process

**Figure 1:**
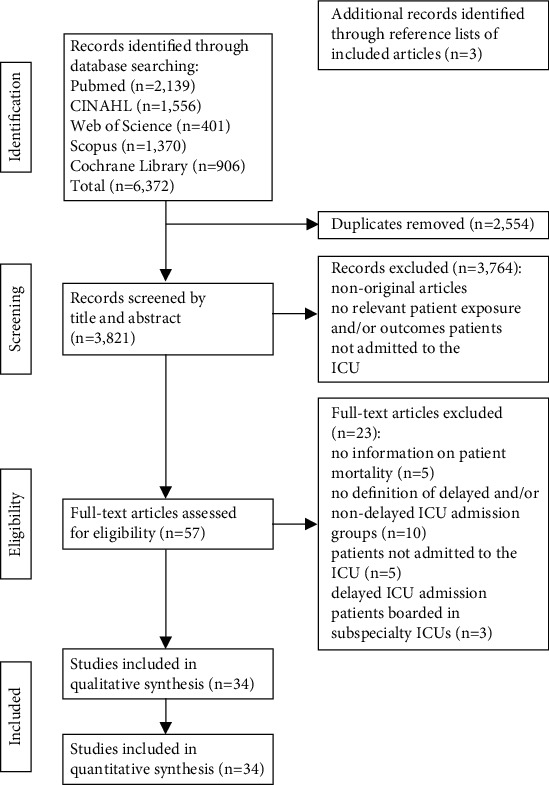
Study selection process: Preferred Reporting Items for Systematic Reviews and Meta-Analyses (PRISMA) flow diagram.

Electronic database searches revealed 6,372 potentially relevant citations (Figure 1). Removal of duplicates, along with screening of titles and abstracts, yielded 54 articles for full-text review. Reference list searches of selected articles revealed three additional articles. Finally, 34 studies (conducted on 34 unique study populations) met eligibility criteria for inclusion in the qualitative and quantitative synthesis.

### 3.2. Study Design, Data Collection, and Bias Assessment

The characteristics of included studies [1, 2, 6, 9–14, 17, 23–46], which were published between 2002 and 2021 and enrolled 356,936 patients in total and 40,348 patients with delayed ICU admission (11.3%), are presented in Table 1. One study had multinational design [10], and nine studies had multicenter design [1, 12, 25, 27, 28, 32, 37, 40, 43], while the other studies had single-center design. Eleven studies used prospective data collection [10, 13, 25, 26, 31, 37–39, 41, 42, 44], and one study used both prospective and retrospective data collection [9], while the other studies used retrospective data collection. According to the NOS score ≥ 6, risk for bias was low in all included studies, while according to the additional criteria used, risk for bias was ≤3 in 20 studies.

### 3.3. Patient Population

Fifteen studies enrolled patients admitted from the ED [1, 2, 9, 11, 23, 24, 27, 30, 31, 33, 38, 39, 41, 43, 45], nine enrolled patients admitted from the wards [6, 13, 28, 29, 32, 35–37, 44], four enrolled postoperative patients [12, 14, 26, 45], and six enrolled patients admitted from various (three or more different) hospital settings. Population size ranged between 91 and 195,428 patients, with 18 studies enrolling <500 patients.

### 3.4. Intervention/Comparison

The nondelayed ICU admission group consisted of patients immediately/directly admitted to the ICU (after the admission decision) in 15 studies [6, 12–14, 17, 25, 29, 32–37, 40, 46] and of patients admitted to the ICU within a time period that was not considered to constitute delay in 19 studies; this time period ranged widely between ≤1 hour and <24 hours. Respectively, the definition of delayed ICU admission was particularly heterogeneous among studies in terms of both immediate/direct ICU admission or not and delay duration, which ranged between ≥1 hour and ≥24 hours. This broad variation rendered impossible the grouping of studies according to the definition of delayed ICU admission. The incidence of delayed ICU admission also ranged widely between 2.1% and 89.5% among studies. Twenty-six studies reported the presence or absence of significant differences in patient characteristics between the delayed and nondelayed ICU admission groups.

### 3.5. Outcome: Qualitative Synthesis

The findings of the included studies are presented in Table 2. Hospital mortality was used as a patient outcome in 27 studies, ICU mortality in 13 studies, and 28-day and 30-day mortality in two studies, while 60-day, 90-day, and 21-ventilator-day mortality were used in one study each. Univariate associations between delayed ICU admission and mortality were reported in 33 studies. In total, unadjusted mortality was significantly higher in the delayed ICU admission group compared to the nondelayed group in 22 studies, specifically hospital mortality in 18 studies [1, 6, 9, 12, 14, 24, 28–32, 35, 36, 39, 40, 43–45], ICU mortality in nine studies [1, 9, 12, 13, 26, 27, 31, 35, 40], 30-day mortality in two studies [12, 17], and 60-day and 90-day mortality in one study each [6, 17]. In 11 studies [2, 10, 11, 23, 33, 34, 37, 38, 41, 42, 46], no significant differences in unadjusted mortality were detected between the delayed and nondelayed ICU admission groups.

Multivariate associations between delayed ICU admission and mortality were evaluated in 22 studies. In three studies [13, 27, 30], delayed ICU admission was entered in the multivariate analysis as a continuous variable (e.g., hours of delay), and no comparisons between the delayed and nondelayed ICU admission groups were reported. In another study [28], although delayed ICU admission was reported not to be associated with significantly higher mortality, adjusted OR and 95% CIs were not provided. In the remaining 18 studies, delayed ICU admission was independently associated with significantly higher mortality in 12 studies, specifically with hospital mortality in eight studies [1, 6, 9, 24, 25, 31, 36, 45], with ICU mortality in two studies [14, 24], and with 28-day, 30-day, 60-day, and 21-ventilator-day mortality in one study each [6, 11, 12, 37]. Delayed ICU admission was not associated with significantly higher adjusted mortality in six studies [10, 17, 23, 33, 34, 39].

### 3.6. Outcome: Quantitative Synthesis

**Figure 2:**
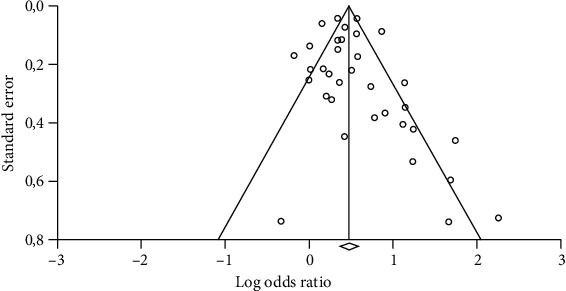
Funnel plot for the assessment of publication bias among studies that reported odds ratios (unadjusted or adjusted) for mortality according to delayed intensive care unit admission. Circles represent odds ratios coming from published studies.

Quantitative synthesis included unadjusted ORs for mortality from 16 studies and adjusted ORs for mortality from 18 studies according to comparisons between delayed and nondelayed ICU admission groups. Overall, in 24 studies, delayed ICU admission was associated with significantly higher mortality. Despite the relative right-sided predominance of study distribution in the funnel plot (Figure 2), Egger's test did not detect significant publication bias (*t* value 1.26, 95% CI -0.40 to 1.72, two-tailed *p* = 0.216).

**Figure 3:**
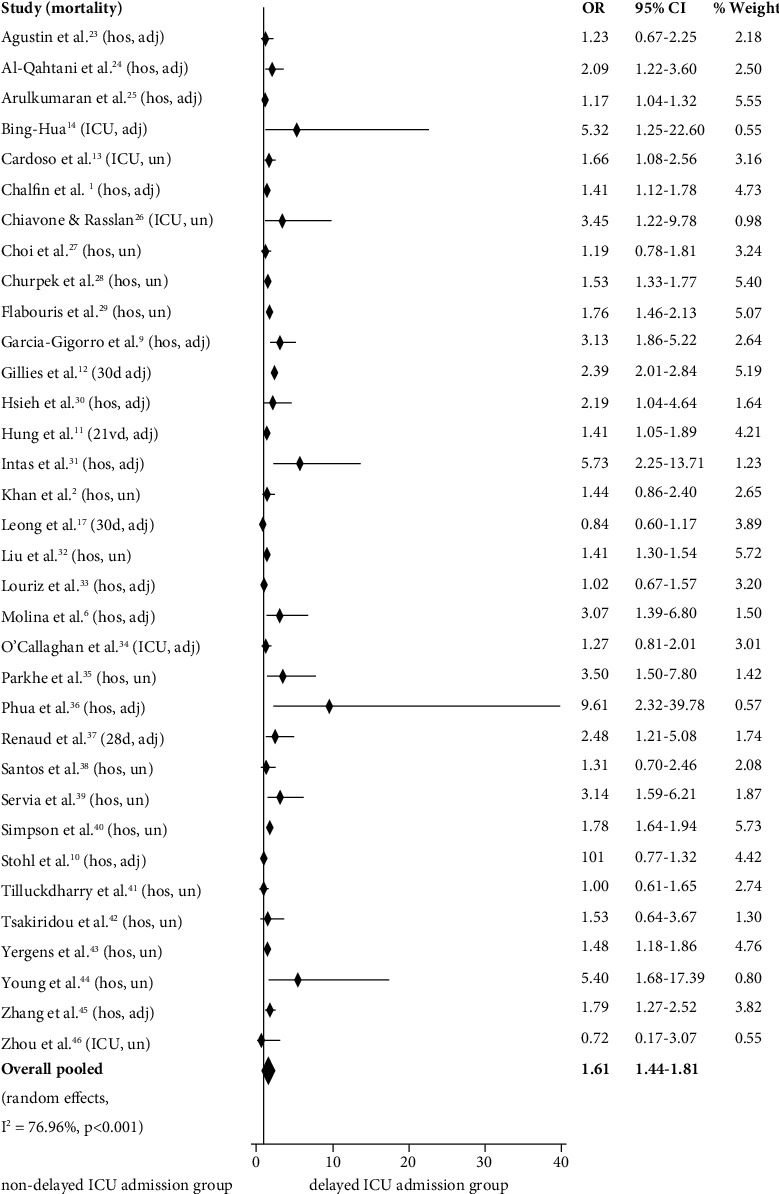
Forest plot depicting individual and pooled odds ratios for mortality with 95% confidence intervals according to delayed intensive care unit admission. OR: odds ratio; CI: confidence interval; hos: hospital; ICU: intensive care unit; 30 d: 30-day; 28 d: 28-day; 21 vd: 21-ventilator-day; adj: adjusted; un: unadjusted.

Pooled OR for mortality was 1.61 (95% CI 1.44-1.81), indicating that delayed ICU admission was associated with significantly higher mortality (Figure 3). *I*^2^ statistic was 66.96%, indicating high heterogeneity among studies. Subgroup and sensitivity analyses are presented in Table 3. In all cases, delayed ICU admission was associated with significantly higher mortality according to the pooled ORs. A remarkably higher pooled OR for mortality was identified for studies in which postoperative patients were included (2.44, 95% CI 1.49-4.01).

### 3.7. Quality of Evidence

According to the GRADE criteria, the starting rating of the quality of evidence for the estimation of pooled OR for mortality was the moderate level, since the included studies had observational design. This was downgraded by one point due to the high inconsistency among individual OR and 95% CI estimates, which were particularly broad and ranged between 0.63 and 39.78, as well as due to the high interstudy heterogeneity. Precision was satisfactory, since the 95% CI around the estimate of the effect of delayed ICU admission was sufficiently narrow, and large numbers of studies and patients were included. Risk for bias was low in most studies (according to the NOS and additional criteria used). Indirectness was not present, since all studies compared the outcomes of interest in the population of interest. No publication bias was identified. Overall, starting rating was downgraded by one point, and this meta-analysis was rated to have low quality of evidence (⊕⊕OO) for a 95% CI of 1.44 to 1.81 (Table 4). This means that the true effect might be markedly different from the present estimate of effect, and further research is likely to have an important impact on this effect.

## 4. Discussion

### 4.1. Summary of Evidence

Considering the high incidence of delayed patient admission to the ICU worldwide and its importance for administrative and therapeutic purposes, this systematic review and meta-analysis summarized the evidence between the association of delayed ICU admission and mortality. In most studies, delayed ICU admission was associated with significantly higher mortality, both unadjusted and adjusted for confounding factors. Overall, quantitative synthesis of findings indicated a significant increase in the odds for mortality by 61% when ICU admission was delayed.

Included studies differed significantly with regard to the definitions of delayed and nondelayed ICU admission, patient populations enrolled, and mortality terms used, which possibly accounted for the wide variation in the incidence of delayed ICU admission and substantial heterogeneity identified. Sensitivity analysis did not reveal remarkable differences in the ORs for mortality according to the definitions of nondelayed ICU admission or mortality terms used; however, the analysis was impossible to include other important differences, such as the delay duration of ICU admission. Despite high heterogeneity, the lack of an internationally accepted definition of delayed ICU admission reflects its inevitably subjective nature and should not preclude aggregation of study findings. Determination of delayed ICU admission can currently be based only on the clinical judgement and experience of the attending physicians, in terms of when patients need to be transferred to the ICU, and which duration of admission delay should be considered clinically important for particular patient populations and healthcare systems with different levels of critical care provision outside the ICU [30, 37, 47].

Unequal distribution of patient and disease characteristics between patients with delayed and nondelayed ICU admission may affect risk for death and confound the association between delayed ICU admission and mortality; thus, individual mortality risk needs to be adjusted. Considering that the priority for ICU admission is given to patients expected to benefit more from critical care, patients with delayed ICU admission have been reported to be older and have higher clinical severity and more comorbidities [13, 14, 24, 28], which might have contributed to their higher mortality. On the other hand, the sickest patients with more rapid clinical decline are generally admitted sooner to the ICU [10, 48]. Despite this controversy, sensitivity analysis indicated only a slight difference between pooled ORs for adjusted and unadjusted mortality, which means that significantly higher mortality associated with delayed ICU admission could not be attributed to the higher individual mortality risk.

Causality in the association between delayed ICU admission and increased mortality is supported by its plausibility. Critically ill patients are particularly susceptible to the adverse effects of omitted or delayed care. Therefore, elements of care which could act as mediators between delayed ICU admission and adverse patient outcome include nurse understaffing, delay in the initiation of time-sensitive treatment (e.g., vasoactive and antibiotic drugs and respiratory support), inadequate training and lack of attention of physicians resulting in delayed patient evaluation and diagnostic testing, unavailability of the multidisciplinary team (e.g., pharmacists and respiratory therapists), increased incidence of errors, and the lack of standardized care that would promote recovery from critical illness (e.g., with regard to delirium prevention and sepsis treatment) [30, 49, 50]. These presumed mediators could also explain the variation in mortality rates reported, considering that the provision of critical care treatment prior to ICU admission is expected to differ considerably among studies and be either timely or delayed. In addition, diverse patient populations can be aggravated by delays in different elements of care, e.g., in initiating early goal-directed antibiotic treatment in septic patients and in detecting hypoxemia in postoperative patients [24, 43].

Homogeneity of groups studied in subgroup analysis was limited. For example, admission from the wards included patients who stayed in the wards for a long time period, those transferred temporarily to the ward from the ED until an ICU bed was available, and those admitted initially to the ward and then to the ICU due to critical deterioration. Likewise, postoperative patients were boarded either in the PACU, surgical unit, or other non-ICU settings. Despite these differences, subgroup analysis revealed a remarkably higher OR for mortality for critically ill postoperative patients with delayed ICU admission. A possible explanation for this finding could be the failure-to-rescue, which refers to patient death after complications that could have been amenable to treatment [51]. Postoperative complications exceed 30% in patients with significant comorbidities; thus, delays in their detection and treatment can be crucial [17, 52]. Furthermore, the personnel of non-ICU settings is expected to provide suboptimal care to the critically ill due to their limited experience and dual focus on both postoperative and ICU overflow patients [53]. This combination of high risk for complications and difficulty to initiate timely life-saving interventions could account for the higher mortality of postoperative critically ill patients.

### 4.2. Limitations and Strengths

There were several limitations that need to be identified. High interstudy heterogeneity is the most important; thus, both quantitative synthesis of study findings and lack of the detection of publication bias should be interpreted with caution. A second limitation of particular importance was that the included studies are susceptible to bias due to their observational design, mainly treatment selection and confirmation bias. Third, searches were conducted in only five electronic databases; therefore, other updated information sources were not covered. Although the articles indexed in these databases are considered to be of satisfactory methodological quality, metabias cannot be excluded. Fourth, most studies had single-center design and used retrospective data collection, which could limit generalizability of their findings. Fifth, only 23 studies reported adjusted associations between delayed ICU admission and mortality; even for them, residual confounding cannot be excluded, since multivariate regression can limit but not eliminate confounding effects. Sixth, the conduction of sensitivity analysis according to the definition of delayed ICU admission was not possible. Seventh, trial sequential analysis, which would have provided more information on the precision and certainty of the present findings, was not conducted. Therefore, the possibility that some positive findings were attributed to a random error rather than the true effects of delayed ICU admission cannot be excluded.

This systematic review and meta-analysis has also remarkable strengths. First, 34 original studies and a relatively large number of patients were included, which ensures satisfactory statistical power. Second, these studies included data from many countries, which adds to the generalizability of the present findings. Third, all studies had high methodological quality according to NOS and most of them demonstrated low risk for bias according to the criteria used.

### 4.3. Implications for Clinical Practice and Future Research

Considering that delayed ICU admission has the potential to contribute to adverse patient outcomes, how could this contribution be minimized? Should more ICU beds become available or should non-ICU settings be more prepared for treating the critically ill? In our opinion, both are equally necessary. The high incidence of delayed ICU admission and the continuous presence of ICU overflow patients in non-ICU hospital settings confirm the need for more ICU beds. At the same time, no matter how many ICU beds are available, it seems doubtful whether their supply could always cover their demand. The current COVID-19 pandemic has led to a global outbreak of respiratory distress and, subsequently, to an unprecedented demand for mechanical ventilation and critical care. To prevent ICUs from being overwhelmed, many countries created new temporary ICU beds from the existing non-ICU ones [54].

However, the initiation of therapeutic management of the critically ill should not depend on the time of their ICU admission. Instead, the operation of more ICU beds is recommended to be combined with the so-called “critical care without walls” [8], which means that the concept of geographically isolated ICUs should be replaced by the expansion of critical care specialty wherever critical illness occurs [55]. This expansion is based on the systematic training of medical and nursing staff of the departments commonly used for boarding the critically ill to develop proficiency in critical care issues. Through this training, optimal care can be provided timely for the acute phase treatment of critically ill patients, so that delays in ICU admission are not translated into delays in the provision of critical care treatment.

A recommended issue for future research would be the evaluation of different cutoff points for delayed ICU admission of critically ill adults, to assess the association between delay duration and adverse patient outcomes. Instead of using a single arbitrary definition for the delayed ICU admission, the conduction of such studies will allow the determination of the exact duration of clinically important delay, as well as of the “golden hour” for ICU admission with regard to diverse critical conditions and patient populations. In addition, the conduction of survival analysis is suggested for modelling time duration after ICU admission with probability of patient death. More research is also needed on postoperative patients, since the number of respective studies was small and these patients were boarded in different non-ICU settings. Since the odds ratio for mortality was found to be remarkably higher for postoperative critically ill adults, the investigation of whether this population benefits from early ICU admission after surgery seems to be particularly important.

## 5. Conclusions

Delayed ICU admission was found to be associated with significantly higher mortality of adult patients considered to need critical care. This finding, along with reported delays and omissions in critical care treatment which can act as mediators for increased mortality when delayed ICU admission occurs, increases the possibility that delayed ICU admission can contribute, to some extent, to higher mortality of critically ill patients. Nevertheless, this explanation should be seen with caution since observational study design cannot establish causality, quality of evidence was low, and the association between delayed ICU admission and mortality could be confounded by treatment selection bias. In this context, increasing the availability of ICU beds needs to be combined with the prompt initiation of critical care treatment in settings commonly used for boarding the critically ill. Especially in the era of the COVID-19 pandemic, during which the increased demand for ICU beds is expected to be followed by an additional increase in the incidence of delayed ICU admission, an imperative need is identified for treatment delays to be prevented, or at least minimized, so that the best possible patient outcomes are ensured.

## Figures and Tables

**Table 1:** Characteristics and assessment of risk for bias of included studies.

Author (year)	Study design/country	Study population	Non-DA/DA group/incidence of DA	Significant differences in patient characteristics between non-DA and DA groups	NOS^∗^/RFB^∗∗^
Agustin et al. [23] (2017)	Retrospective, single-center/US	287 ED pts with severe sepsis and septic shock	Pts with ED LOS < 6 hrs (150)/pts with ED LOS ≥ 6 hrs (137)/47.7%	DA pts had lower initial lactate level	9/4
Al-Qahtani et al. [24] (2017)	Retrospective, single-center/Saudi Arabia	940 ED pts	Pts with ED LOS < 6 hrs (227)/pts with ED LOS between 6 and 24 hrs (358) and >24 hrs (355)/75.9%	DA pts were older and had longer duration of mechanical ventilation	9/3
Arulkumaran et al. [25] (2017)	Prospective, multicenter/UK	195,428 medical/surgical ward, obstetric/intermediate care areas, ED, and OR pts	Pts immediately admitted (187,133)/pts remaining outside ICU for ≤4 hrs (6,198) and >4 hrs (2,097)/4.2%	Not reported	8/1
Bing-Hua [14] (2014)	Retrospective, single-center/China	2,279 postoperative pts	Pts immediately admitted (2,094)/pts boarding in PACU for ≤2, 2-4, 4-6, and >6 hrs (185)/8.1%	DA pts were older and more likely to have diabetes and chronic lung disease	7/3
Cardoso et al. [13] (2011)	Prospective, single-center/Brazil	401 ED and general ward pts	Pts immediately admitted (125)/pts admitted from wards after ≤72 hrs (276)/68.8%	DA pts had more comorbidities	9/2
Chalfin et al. [1] (2007)	Retrospective, multicenter/US	50,322 ED pts	Pts with ED LOS < 6 hrs (49,286)/pts with ED LOS ≥ 6 hrs (1,036)/2.1%	No differences were noted	8/3
Chiavone and Rasslan [26] (2005)	Prospective, single-center/Brazil	94 postoperative pts after emergency surgery	Pts boarding in surgical unit for ≤12 hrs after the end of surgery (23)/pts boarding in surgical unit for >12 hrs (71)/75.5%	No differences were noted	6/4
Choi et al. [27] (2021)	Retrospective, multicenter/Republic of Korea	439 ED pts > 65 years with infectious diseases	Pts with ED LOS ≤ 6 hrs (179)/pts with ED LOS > 6 hrs (260) and >24 hrs (86)/59.2% and 19.6%, respectively	Not reported	8/4
Churpek et al. [28] (2016)	Retrospective, multicenter/US	3,789 medical/surgical ward pts	Pts admitted within 6 hrs (2,055)/pts admitted after ≥6 hrs (1,734)/45.7%	DA pts were older	8/2
Flabouris et al. [29] (2012)	Retrospective, single-center/Australia	21,960 ED and general ward pts	Pts directly admitted from ED (21,481)/pts admitted from general wards (479)/2.2%	DA pts had higher clinical severity	8/3
García-Gigorro et al. [9] (2017)	Prospective and retrospective, single-center/Spain	269 ED pts	Pts with ED LOS ≤ 5 hrs (140)/pts with ED LOS > 5 hrs (129)/48.0%	Not reported	6/4
Gillies et al. [12] (2017)	Retrospective, multicenter/UK	13,591 postoperative pts (excluding cardiac surgery and neurosurgery)	Pts immediately admitted after surgery (1,116)/pts admitted from non-ICU settings after ≤7 days (12,475)/89.5%	DA pts were older and had higher operative severity and emergency surgical status	9/1
Hsieh et al. [30] (2017)	Retrospective, single-center/Taiwan	267 ED pts with acute respiratory failure	Pts with ED LOS ≤ 1 hr (196)/pts with ED LOS > 1 hr (71)/26.6%	Not reported	7/5
Hung et al. [11] (2014)	Retrospective, single-center/Taiwan	1,242 nontrauma ED pts with ventilatory support	Pts with ED LOS ≤ 4 hrs (337)/pts with ED LOS > 4 hrs (905)/72.9%	Not reported	7/4
Intas et al. [31] (2012)	Prospective, single-center/Greece	200 intubated ED pts	Pts with ED LOS < 6 hrs (60)/pts with ED LOS ≥ 6 hrs (140)/70.0%	More DA pts were female and medical, had higher age, were more likely to manifest fever, and received more medicines	8/3
Khan et al. [2] (2016)	Retrospective, single-center/Pakistan	325 ED pts	Pts with ED LOS ≤ 6 hrs (164)/pts with ED LOS > 6 hrs (161)/49.5%	DA pts had lower GCS scores, were less likely to have history of endocrine disease, and more likely to have history of CNS disease	8/4
Leong et al. [17] (2019)	Retrospective, single-center/US	4,282 ED, OR, and ward pts	Pts directly admitted from ED or OR (3,862)/pts admitted from wards after ≤24 hrs (420)/9.8%	No differences were noted	8/3
Liu et al. [32] (2012)	Retrospective, multicenter/US	36,298 ED and ward pts	Pts directly admitted from ED (29,929)/pts admitted from wards after ≤24 hrs (6,369)/17.5%	Not reported	7/3
Louriz et al. [33] (2012)	Retrospective, single-center/Morocco	256 ED pts	Pts immediately admitted from ED (110)/pts admitted from wards (146)/57.0%	DA pts were older and had more comorbidities	8/4
Molina et al. [6] (2014)	Retrospective, single-center/Singapore	698 ED and ward pts	Pts directly admitted from ED (490)/pts admitted from wards after ≤24 hrs (208)/29.8%	DA pts were older and less likely to undergo resuscitation or intubation in ED	9/3
O'Callaghan et al. [34] (2012)	Retrospective, single-center/UK	1,609 ED, OR, and ward pts	Pts immediately admitted from ED (1,460)/pts admitted from ED, OR, or wards after >3 hrs (149)/9.3%	DA pts were more likely to have respiratory failure	8/3
Parkhe et al. [35] (2002)	Retrospective, single-center/Australia	122 ED and ward pts	Pts directly admitted from ED (99)/pts admitted from wards after ≤24 hrs (23)/18.9%	DA pts were older, had higher clinical severity, and were more likely to have history of cardiac, respiratory, and gastrointestinal disease	7/4
Phua et al. [36] (2010)	Retrospective, single-center/Singapore	103 ED and general ward pts	Pts directly admitted from ED (54)/pts admitted from general wards after ≤72 hrs (49)/47.6%	DA pts were older and less likely to have unstable vital signs and had better mental status	8/3
Renaud et al. [37] (2009)	Prospective, multicenter/US, France	453 ED and medical ward pts	Pts directly admitted from ED (315)/pts admitted from medical wards after 2-3 days (138)/30.5%	DA pts were more likely to have cardiovascular disease or diabetes and less likely to have abnormal mental status, tachycardia, tachypnea, acidosis, and multilobar infiltrates	9/1
Santos et al. [38] (2020)	Prospective, single-center/Brazil	206 ED pts	Pts with ED LOS < 637 min (65)/pts with ED LOS ≥ 637 min (141)/67.5%	DA pts were older and more likely to need assistance	7/4
Serviá et al. [39] (2012)	Prospective, single-center/Spain	243 ED pts with severe trauma	Pts with ED LOS ≤ 120 min (122)/pts with ED LOS > 120 min (121)/49.8%	DA pts were older and less likely to manifest shock, be mechanically ventilated, and need blood transfusion and had higher injury severity	8/3
Simpson et al. [40] (2005)	Retrospective, multicenter/UK	12,268 ED, ward, and intermediate care areas pts	Pts directly admitted from ED (9,389)/pts admitted from wards or intermediate care areas (2,879)/23.5%	DA pts were older and more likely to have severe past medical history	8/3
Stohl et al. [10] (2019)	Prospective, multinational	3,175 pts of any hospital setting	Pts admitted within 4 hrs (2,754)/pts admitted after ≥4 hrs (421)/13.3%	Not reported	8/2
Tilluckdharry et al. [41] (2005)	Prospective, single-center/US	443 ED pts	Pts with ED LOS < 24 hrs (339)/pts with ED LOS ≥ 24 hrs (104)/23.5%	No differences were noted	8/5
Tsakiridou et al. [42] (2018)	Prospective, single-center/Greece	100 pts of any hospital setting with VAP	Pts admitted within 24 hrs (68)/pts admitted after ≥24 hrs (32)/32.0%	DA pts were more likely to be previously hospitalized and have chronic renal failure and received more antibiotics	7/4
Yergens et al. [43] (2015)	Retrospective, multicenter/Canada	1,770 ED pts with sepsis or severe sepsis	Pts with ED LOS ≤ 7 hrs (488)/pts with ED LOS > 7 hrs (1,282)/72.4%	DA pts were older and had higher triage level	8/3
Young et al. [44] (2003)	Prospective, single-center/US	91 ward pts with noncardiac diagnoses	Pts admitted within 4 hrs (35)/pts admitted after ≥4 hrs (56)/61.5%	No differences were noted	8/3
Zhang et al. [45] (2019)	Retrospective, single-center/China	1,997 ED pts with sepsis	Pts with ED LOS < 6 hrs (1,306)/pts with ED LOS ≥ 6 hrs (691)/34.6%	Not reported	8/4
Zhou et al. [46] (2015)	Retrospective, single-center/China	989 postoperative neurosurgical pts	Pts immediately admitted from OR (937)/pts boarding in PACU for ≤2 and >2 hrs (52)/5.3%	DA pts were less likely to be neurooncological	6/4

ICU: intensive care unit; ED: emergency department, PACU: postanesthesia care unit; OR: operating room; LOS: length of stay; GCS: Glasgow Coma Scale; CNS: central nervous system; VAP: ventilator-associated pneumonia; DA: delayed ICU admission; NOS: Newcastle-Ottawa Scale; RFB: risk for bias according to additional criteria; pts: patients; hr: hour; min: minutes. ^∗^Score ranging from 0 to 9; the higher the score, the lower the risk for bias. ^∗∗^Score ranging from 0 to 7; the higher the score, the higher the risk for bias.

**Table 2:** Findings of included studies.

Author (year)	Unadjusted mortality (univariate associations)	Adjusted mortality (multivariate associations)
Agustin et al. [23] (2017)	No significant difference in hospital mortality between pts with ED LOS ≥ 6 hrs and those with ED LOS < 6 hrs: 24.7% vs. 22.6%, OR 1.12, 95% CI 0.65-1.93, *p* = 0.685	ED LOS ≥ 6 hrs was not associated with higher hospital mortality: OR 1.23, 95% CI 0.67-2.25, *p* = 0.510
Al-Qahtani et al. [24] (2017)	Pts with ED LOS between 6 and 24 hrs and >24 hrs had higher hospital mortality than those with ED LOS < 6 hrs: 29.1% and 37.2% vs. 22.5%, respectively, *p* < 0.001 No significant difference in ICU mortality between pts with ED LOS between 6 and 24 hrs and >24 hrs and those with ED LOS < 6 hrs: 21.8% and 25.2% vs. 18.1%, *p* = 0.130	ED LOS > 24 hrs was independently associated with higher hospital mortality: OR 2.09, 95% CI 1.22-3.60, *p* = 0.007 ED LOS > 24 hrs was independently associated with higher ICU mortality: OR 1.90, 95% CI 1.02-3.54, *p* = 0.040
Arulkumaran et al. [25] (2017)	Not reported	Remaining outside ICU for ≤4 hrs and >4 hrs were independently associated with higher hospital mortality: OR 1.08, 95% CI 1.01-1.17, and OR 1.17, 95% CI 1.04-1.32, *p* = 0.004, respectively
Bing-Hua [14] (2014)	No significant difference in ICU mortality between pts immediately admitted to ICU and those boarding in PACU: 8.6% vs. 6.7%, *p* = 0.311 Pts boarding in PACU for >6 hrs had higher hospital mortality than those immediately admitted to ICU: 15.4% vs. 6.7%, *p* < 0.001	Boarding in PACU for >6 hrs was independently associated with higher ICU mortality: OR 5.32, 95% CI 1.25-22.60, *p* = 0.024
Cardoso et al. [13] (2011)	Pts not immediately admitted to the ICU had higher ICU mortality than immediately admitted ones: 50.0% vs. 37.6%, OR 1.66, 95% CI 1.08-2.56, *p* < 0.01	Each hr of delayed ICU admission was independently associated with 1.0% increase in hospital mortality and 1.5% increase in ICU mortality: HR 1.01, 95% CI 1.00-1.02, *p* = 0.014, and HR 1.02, 95% CI 1.01-1.02, *p* = 0.001, respectively
Chalfin et al. [1] (2007)	Pts with ED LOS > 6 hrs had higher hospital and ICU mortality than those with ED LOS < 6 hrs: 17.4% vs. 12.9%, *p* < 0.001, and 10.7% vs. 8.4%, *p* = 0.009, respectively	ED LOS > 6 hrs was independently associated with higher hospital mortality: OR 1.41, 95% CI 1.12-1.78, *p* = 0.004
Chiavone and Rasslan [26] (2005)	Pts with PACU LOS > 12 hrs had higher ICU mortality than those with PACU LOS ≤ 12 hrs: 54.9% vs. 26.1%, OR 3.45, 95% CI 1.22-9.78, *p* = 0.018	Not reported
Choi et al. [27] (2021)	No significant difference in hospital mortality between pts with ED LOS > 6 hrs and those with ED LOS ≤ 6 hrs: 31.5% vs. 27.9%, OR 1.19, 95% CI 0.78-1.81, *p* = 0.418 Pts with ED LOS > 24 hrs had higher ICU mortality than those with ED LOS ≤ 24 hrs: 41.9% vs. 27.2%, OR 1.93, 95% CI 1.18-3.14, *p* = 0.008	ED LOS (as continuous variable) was independently associated with higher hospital mortality: OR 1.01, 95% CI 1.00-1.02, *p* = 0.039
Churpek et al. [28] (2016)	Pts admitted to ICU after >6 hrs had higher hospital mortality than those admitted within 6 hrs: 33.2% vs. 24.5%, OR 1.53, 95% CI 1.33-1.77, *p* < 0.001	Each hr of delayed ICU admission was independently associated with 3.0% increase in hospital mortality, *p* < 0.001
Flabouris et al. [29] (2012)	Pts initially admitted to general wards had higher hospital mortality than those directly admitted to ICU: 34.9% vs. 23.3%, OR 1.76, 95% CI 1.46-2.13, *p* < 0.01	Not reported
García-Gigorro et al. [9] (2017)	Pts with ED LOS > 5 hrs had higher hospital and ICU mortality than those with ED LOS ≤ 5 hrs: 21.7% vs. 8.6%, *p* = 0.003, and 17.8% vs. 7.1%, *p* = 0.006, respectively	ED LOS > 5 hrs was independently associated with higher hospital mortality: OR 3.13, 95% CI 1.86-5.22
Gillies et al. [12] (2017)	Pts admitted to ICU after ≤7 days had higher hospital, perioperative (30-day), and ICU mortality than immediately admitted ones: 24.3% vs. 14.0%, *p* < 0.01, 20.9% vs. 12.1%, *p* < 0.01, and 15.2% vs. 6.9%, *p* < 0.01, respectively	Admission to ICU after ≤7 days was independently associated with higher perioperative (30-day) mortality: OR 2.39, 95% CI 2.01-2.84, *p* < 0.01
Hsieh et al. [30] (2017)	Pts with ED LOS > 1 hr had higher hospital mortality than those with ED LOS ≤ 1 hr: 84.5% vs. 71.4%, OR 2.18, 95% CI 1.07-4.45, *p* = 0.03	ED LOS > 1 hr was independently associated with higher hospital mortality: OR 2.19, 95% CI 1.04-4.64, *p* = 0.04
Hung et al. [11] (2014)	No significant difference in 21-ventilator-day mortality between pts with ED LOS > 4 hrs and those with ED LOS ≤ 4 hrs: OR 1.17, 95% CI 0.98-1.39, *p* = 0.093	ED LOS > 4 hrs was independently associated with higher 21-ventilator-day mortality: OR 1.41, 95% CI 1.05-1.89, *p* = 0.024
Intas et al. [31] (2012)	Pts with ED LOS ≥ 6 hrs had higher hospital and ICU mortality than those with ED LOS < 6 hrs: 62.9% vs. 46.7%, *p* = 0.001, and 43.5% vs. 22.2%, *p* < 0.001, respectively	ED LOS ≥ 6 hrs was independently associated with higher hospital mortality: OR 5.73, 95% CI 2.25-13.71, *p* < 0.001
Khan et al. [2] (2016)	No significant difference in hospital mortality between pts with ED LOS > 6 hrs and those with ED LOS ≤ 6 hrs: 27.3% vs. 20.7%, OR 1.44, 95% CI 0.86-2.40, *p* = 0.160	Not reported
Leong et al. [17] (2019)	Pts admitted to ICU after ≤24 hrs had higher 30-day and 90-day mortality than directly admitted ones: 15.0% vs. 9.9%, *p* = 0.03, and 20% vs. 13%, *p* = 0.005, respectively	Admission to ICU after ≤24 hrs was not associated with higher 30-day mortality: OR 0.84, 95% CI 0.60-1.17, *p* = 0.296
Liu et al. [32] (2012)	Pts admitted to ICU after ≤24 hrs had higher hospital mortality than directly admitted ones: 11.6% vs. 8.5%, OR 1.41, 95% CI 1.30-1.54, *p* < 0.01	Not reported
Louriz et al. [33] (2012)	No significant difference in hospital mortality between pts admitted from wards and immediately admitted ones: 43.8% vs. 33.3%, HR 1.11, 95% CI 0.74-1.68, *p* = 0.59	Delayed admission to ICU from wards was not associated with higher hospital mortality: OR 1.02, 95% CI 0.67-1.57, *p* = 0.89
Molina et al. [6] (2014)	Pts admitted to ICU after ≤24 hrs had higher hospital and 60-day mortality than directly admitted ones: 32.2% vs. 27.0%, *p* < 0.01, and 52.3% vs. 43.3%, *p* < 0.01, respectively	Admission to ICU after ≤24 hrs was independently associated with higher hospital and 60-day mortality: OR 3.07, 95% CI 1.39-6.80, and OR 3.09, 95% CI 1.40-6.83, respectively
O'Callaghan et al. [34] (2012)	No significant difference in hospital and ICU mortality between pts admitted to ICU after >3 hrs and directly admitted ones: 36.2% vs. 32.8%, *p* = 0.44, and 26.8% vs. 24.2%, *p* = 0.47, respectively	Admission to ICU after >3 hrs was not associated with higher ICU mortality: OR 1.27, 95% CI 0.81-2.01, *p* = 0.29
Parkhe et al. [35] (2002)	Pts admitted to ICU after ≤24 hrs had higher hospital and ICU mortality than directly admitted ones: 34.8% vs. 14.1%, OR 3.5, 95% CI 1.5-7.8, *p* = 0.044, and 34.8% vs. 9.1%, OR 2.5, 95% CI 1.2-5.2, *p* = 0.007, respectively	Not reported
Phua et al. [36] (2010)	Pts admitted to ICU after ≤72 hrs had higher hospital mortality than directly admitted ones: 51.0% vs. 20.4%, *p* = 0.001	Admission to ICU after ≤72 hrs was independently associated with higher hospital mortality: OR 9.61, 95% CI 2.32-39.78, *p* = 0.002
Renaud et al. [37] (2009)	No significant difference in 28-day mortality between pts admitted to ICU after 2-3 days and directly admitted ones: 19.6% vs. 13.6%, OR 1.54, 95% CI 0.91-2.61, *p* = 0.11	Admission to ICU after 2-3 days was independently associated with higher 28-day mortality: OR 2.48, 95% CI 1.21-5.08, *p* = 0.01
Santos et al. [38] (2020)	No significant difference in hospital and ICU mortality between pts with ED LOS < 637 min and those with ED LOS ≥ 637 min: 30.8% vs. 36.9%, OR 1.31, 95% CI 0.70-2.46, *p* = 0.639, and 24.6% vs. 29.8%, OR 1.30, 95% CI 0.66-2.54, *p* = 0.707, respectively ED LOS (as continuous variable) was not associated with hospital mortality: OR 1.20, 95% CI 0.68-2.13, *p* = 0.527	Not reported
Serviá et al. [39] (2012)	Pts with ED LOS > 120 min had higher hospital mortality than those with ED LOS ≤ 120 min: 28.7% vs. 11.6%, OR 3.14, 95% CI 1.59-6.21, *p* = 0.011	ED LOS > 120 min was not associated with higher hospital mortality (OR not reported)
Simpson et al. [40] (2005)	Pts not directly admitted to ICU had higher hospital and ICU mortality than directly admitted ones: 46.4% vs. 32.7%, OR 1.78, 95% CI 1.64-1.94, *p* < 0.001, and 36.8% vs. 26.2%, OR 1.64, 95% CI 1.50-1.79, *p* < 0.001, respectively	Not reported
Stohl et al. [10] (2019)	No significant difference in 28-day mortality between pts admitted to ICU after ≥4 hrs and those admitted within 4 hrs: 25.2% vs. 29.6%, OR 0.80, 95% CI 0.63-1.01, *p* = 0.06	Admission to ICU after ≥4 hrs was not associated with higher 28-day, hospital, ICU, and 3-month mortality: OR 1.10, 95% CI 0.85-1.43, *p* = 0.45, OR 1.01, 95% CI 0.77-1.32, *p* = 0.94, OR 0.95, 95% CI 0.71-1.26, *p* = 0.71, and OR 1.07, 95% CI 0.84-1.38, *p* = 0.58, respectively
Tilluckdharry et al. [41] (2005)	No significant difference in hospital mortality between pts with ED LOS ≥ 24 hrs and those with ED LOS < 24 hrs: 26.8% vs. 26.9%, OR 1.0, 95% CI 0.61-1.65, *p* = 0.5	Not reported
Tsakiridou et al. [42] (2018)	No significant difference in hospital mortality between pts admitted to ICU after ≥24 hrs and those admitted within 24 hrs: 40.6% vs. 30.9%, OR 1.53, 95% CI 0.64-3.67, *p* = 0.337	Not reported
Yergens et al. [43] (2015)	Pts with ED LOS > 7 hrs had higher hospital mortality than those with ED LOS ≤ 7 hrs: 74.6% vs. 66.4%, OR 1.48, 95% CI 1.18-1.86, *p* = 0.001	Not reported
Young et al. [44] (2003)	Pts admitted after ≥4 hrs had higher hospital mortality than those admitted within 4 hrs: 41.1% vs. 11.4%, OR 5.40, 95% CI 1.68-17.39, *p* = 0.004	Not reported
Zhang et al. [45] (2019)	Pts with ED LOS of 12-24 hrs and >24 hrs had higher hospital mortality than those with ED LOS < 6 hrs: 31.9% and 31.8% vs. 21.4%, *p* < 0.001, respectively	ED LOS of 12-24 hrs and >24 hrs was independently associated with higher hospital mortality: OR 1.82, 95% CI 1.28-2.58, *p* < 0.001, and OR 1.79, 95% CI 1.27-2.52, *p* < 0.001, respectively
Zhou et al. [46] (2015)	No significant difference in ICU mortality between pts immediately admitted to ICU and those boarding in PACU: 5.2% vs. 3.8%, *Ο*R 0.72, 95% CI 0.17-3.07, *p* = 0.681	Not reported

ICU: intensive care unit; ED: emergency department; PACU: postanesthesia care unit; LOS: length of stay; OR: odds ratio; HR: hazard ratio; CI: confidence interval; pts: patients; hr: hour; min: minutes.

**Table 3:** Subgroup and sensitivity analyses: pooled odds ratios for mortality according to delayed intensive care unit admission and 95% confidence intervals.

	Pooled odds ratio	95% confidence interval
*Patient population*		
Patients admitted from the emergency department (*n* = 15)	1.64	1.38-1.94
Patients admitted from the wards (*n* = 9)	1.78	1.49-2.13
Postoperative patients (*n* = 4)	2.44	1.49-4.01
*Definition of nondelayed ICU admission group*		
Patients immediately/directly admitted to the ICU (*n* = 15)	1.62	1.36-1.93
Patients with shorter ICU admission delay than that of the delayed ICU admission group (*n* = 19)	1.63	1.39-1.88
*Mortality*		
Unadjusted (*n* = 16)	1.59	1.42-1.79
Adjusted (*n* = 18)	1.71	1.38-2.12
Hospital (*n* = 25)	1.51	1.49-1.58
ICU (*n* = 11)	1.57	1.27-1.95

**Table 4:** GRADE evidence profile.

Outcome examined (*n* = 34)	Study design	Certainty assessment					Summary of findings				Certainty
		Risk for bias	Inconsistency	Indirectness	Imprecision	Publication bias	DA/non-DA pts (*n*)	Relative effect (95% CI)	Absolute effects (95% CI)		
									DA	Non-DA	
Mortality	Observational cohort (⊕⊕⊕O)	Not serious	Serious	Not serious	Not serious	Not serious	40,348/316,588	OR, 1.61 (1.44, 1.81)	271 per 1,000 (258-284)	163 per 1,000 (154-172)	Low (⊕⊕OO)

OR: odds ratio; CI: confidence interval; DA: delayed ICU admission; pts: patients.

## Data Availability

All data used to support the findings of this study are available from the corresponding author on reasonable request.

## Supplementary Materials

Supplementary Materials Search strings used in electronic databases. PubMed: (delayed admission(tiab) OR admission delay(tiab) OR indirect admission(tiab) OR delayed transfer(tiab) OR boarding(tiab) OR emergency department(tiab)) AND (mortality(tiab) OR outcome(tiab)) AND (intensive care unit(tiab) OR ICU(tiab) OR critically ill(tiab)). CINAHL: “delayed admission OR admission delay OR indirect admission OR delayed transfer OR boarding OR emergency department AND mortality OR outcome AND intensive care unit OR ICU OR critically ill on 2021-09-06 10:00 PM”. Scopus: ((‘delayed admission':ti,ab,kw OR ‘admission delay':ti,ab,kw OR ‘indirect admission':ti,ab,kw OR ‘delayed transfer':ti,ab,kw OR ‘boarding':ti,ab,kw OR ‘emergency department':ti,ab,kw) AND (‘mortality':ti,ab,kw OR ‘outcome':ti,ab,kw) AND (‘intensive care unit':ti,ab,kw OR ‘ICU':ti,ab,kw OR ‘critically ill':ti,ab,kw)). The Cochrane Library: (“delayed admission” or “admission delay” or “indirect admission” or “delayed transfer” or boarding or “emergency department”:ti,ab.kw) and (mortality or outcome:ti,ab,kw) and (“intensive care unit” or ICU or “critically ill”:ti,ab,kw). Web of Science: (“delayed admission” OR “admission delay” OR “indirect admission” OR “delayed transfer” OR boarding OR “emergency department”) AND (mortality OR outcome) AND (“intensive care unit” OR ICU OR “critically ill”).
