# Supplementary material for: Delayed Admission to the Intensive Care Unit and Mortality of Critically Ill Adults: Systematic Review and Meta-analysis
Source: Biomed Res Int. 2022 Feb 7;2022:4083494. doi: 10.1155/2022/4083494 (PMC8822318; doi:10.1155/2022/4083494)
Supplement: Supplementary Materials — Search strings used in electronic databases. PubMed: (delayed admission(tiab) OR admission delay(tiab) OR indirect admission(tiab) OR delayed transfer(tiab) OR boarding(tiab) OR emergency department(tiab)) AND (mortality(tiab) OR outcome(tiab)) AND (intensive care unit(tiab) OR ICU(tiab) OR critically ill(tiab)). CINAHL: “delayed admission OR admission delay OR indirect admission OR delayed transfer OR boarding OR emergency department AND mortality OR outcome AND intensive care unit OR ICU OR critically ill on 2021-09-06 10:00 PM”. Scopus: ((‘delayed admission':ti,ab,kw OR ‘admission delay':ti,ab,kw OR ‘indirect admission':ti,ab,kw OR ‘delayed transfer':ti,ab,kw OR ‘boarding':ti,ab,kw OR ‘emergency department':ti,ab,kw) AND (‘mortality':ti,ab,kw OR ‘outcome':ti,ab,kw) AND (‘intensive care unit':ti,ab,kw OR ‘ICU':ti,ab,kw OR ‘critically ill':ti,ab,kw)). The Cochrane Library: (“delayed admission” or “admission delay” or “indirect admission” or “delayed transfer” or boarding or “emergency department”:ti,ab.kw) and (mortality or outcome:ti,ab,kw) and (“intensive care unit” or ICU or “critically ill”:ti,ab,kw). Web of Science: (“delayed admission” OR “admission delay” OR “indirect admission” OR “delayed transfer” OR boarding OR “emergency department”) AND (mortality OR outcome) AND (“intensive care unit” OR ICU OR “critically ill”). [file 4083494.f1.docx]

**Supplementary material**

**Search strings used in electronic databases**

***Pubmed:*** (delayed admission(tiab) OR admission delay(tiab) OR indirect admission(tiab) OR delayed transfer(tiab) OR boarding(tiab) OR emergency department(tiab)) AND (mortality(tiab) OR outcome(tiab)) AND (intensive care unit(tiab) OR ICU(tiab) OR critically ill(tiab)).

***CINAHL:*** “delayed admission OR admission delay OR indirect admission OR delayed transfer OR boarding OR emergency department AND mortality OR outcome AND intensive care unit OR ICU OR critically ill on 2021-09-06 10:00 PM”.

***Scopus***: ((‘delayed admission’:ti,ab,kw OR ‘admission delay’:ti,ab,kw OR ‘indirect admission’:ti,ab,kw OR ‘delayed transfer’:ti,ab,kw OR ‘boarding’:ti,ab,kw OR ‘emergency department’:ti,ab,kw) AND (‘mortality’:ti,ab,kw OR ‘outcome’:ti,ab,kw) AND (‘intensive care unit’:ti,ab,kw OR ‘ICU’:ti,ab,kw OR ‘critically ill’:ti,ab,kw)).

***The Cochrane Library***: (“delayed admission” or “admission delay” or “indirect admission” or “delayed transfer” or boarding or “emergency department”:ti,ab.kw) and (mortality or outcome:ti,ab,kw) and (“intensive care unit” or ICU or “critically ill”:ti,ab,kw).

***Web of Science:*** (“delayed admission” OR “admission delay” OR “indirect admission” OR “delayed transfer” OR boarding OR “emergency department”) AND (mortality OR outcome) AND (“intensive care unit” OR ICU OR “critically ill”).
